# Augmented expression of cardiac ankyrin repeat protein is induced by pemetrexed and a possible marker for the pemetrexed resistance in mesothelioma cells

**DOI:** 10.1186/s12935-017-0493-8

**Published:** 2017-12-11

**Authors:** Yiyang Qin, Ikuo Sekine, Mengmeng Fan, Yuichi Takiguchi, Yuji Tada, Masato Shingyoji, Michiko Hanazono, Naoto Yamaguchi, Masatoshi Tagawa

**Affiliations:** 1 Division of Pathology and Cell Therapy, Chiba Cancer Center Research Institute, 666-2 Nitona, Chuo-ku, Chiba, 260-8717 Japan; 2 Laboratory of Molecular Cell Biology, Graduate School of Pharmaceutical Sciences, Chiba University, Chiba, Japan; 3 Department of Medical Oncology, Faculty of Medicine, University of Tsukuba, Ibaraki, Japan; 4 Department of Medical Oncology, Graduate School of Medicine, Chiba University, Chiba, Japan; 5 Department of Respirology, Graduate School of Medicine, Chiba University, Chiba, Japan; 6 Division of Respirology, Chiba Cancer Center, Chiba, Japan; 7 Department of Molecular Biology and Oncology, Graduate School of Medicine, Chiba University, Chiba, Japan

**Keywords:** Mesothelioma, PEM resistance, Cardiac ankyrin repeat protein, Biomarker

## Abstract

**Background:**

Pemetrexed (PEM) is an anti-cancer agent targeting DNA and RNA synthesis, and clinically in use for mesothelioma and non-small cell lung carcinoma. A mechanism of resistance to PEM is associated with elevated activities of several enzymes involved in nucleic acid metabolism.

**Methods:**

We established two kinds of PEM-resistant mesothelioma cells which did not show any increase of the relevant enzyme activities. We screened genes enhanced in the PEM-resistant cells with a microarray analysis and confirmed the expression levels with Western blot analysis. A possible involvement of the candidates in the PEM-resistance was examined with a WST assay after knocking down the expression with si-RNA. We also analyzed a mechanism of the up-regulated expression with agents influencing AMP-activated protein kinase (AMPK) and p53.

**Results:**

We found that expression of cardiac ankyrin repeat protein (CARP) was elevated in the PEM-resistant cells with a microarray and Western blot analysis. Down-regulation of CARP expression with si-RNA did not however influence the PEM resistance. Parent and PEM-resistant cells treated with PEM increased expression of CARP, AMPK, p53 and histone H2AX. The CARP up-regulation was however irrelevant to the *p53* genotypes and not induced by an AMPK activator. Augmented p53 levels with nutlin-3a, an inhibitor for p53 degradation, and DNA damages were not always associated with the enhanced CARP expression.

**Conclusions:**

These data collectively suggest that up-regulated CARP expression is a potential marker for development of PEM-resistance in mesothelioma and that the PEM-mediated enhanced expression is not directly linked with immediate cellular responses to PEM.

**Electronic supplementary material:**

The online version of this article (10.1186/s12935-017-0493-8) contains supplementary material, which is available to authorized users.

## Background

Malignant mesothelioma, developed in the pleural cavity, is highly resistant to a number of therapeutics and prognosis of the patients remains poor. A combination of pemetrexed (PEM) and cisplatin (CDDP) is the first-line chemotherapy regimen for more than a decade [1]. No second-line regimen is yet established and molecular target agents did not produce better outcomes than the first-line agents [2]. Drug resistance to the anti-cancer agents, often developed in a number of the patients, is one of crucial issues in clinical settings and overcoming the resistance is important in terms of the efficacy of chemotherapy. Machinery of CDDP resistance have been investigated in many types of cancer [3], but that an underlying mechanism to PEM resistance in mesothelioma is unclear [4].

Pemetrexed is a potent DNA and RNA synthesis inhibitor and is reported to target three kinds of enzymes involved in purine and pyrimidine synthesis, thymidylate synthase (TS), dihydrofolate reductase (DHFR) and glycinamide ribonucleotide formyltransferase (GARFT) [5]. An expression level of TS in tumors was linked to sensitivity to PEM, but contribution of the other two enzymes to PEM-resistance remained unsettled [6]. Previous studies with lung cancer patients showed that PEM resistance was associated with an TS-linked enzyme such as uracil-DNA glycosylase [7], and with expression of growth signal molecules including the epidermal growth factor receptor and p38 MAP kinase [8, 9]. PEM also inhibits an action of the aminoimidazolecarboxamide ribonucleotide formyltransferase, involved in a folate-mediated de novo purine synthesis, and consequently 5-amino-4-imidazolecarboxamide ribotide (ZMP) was accumulated in PEM-treated cells [10]. The intermediate molecules activates the AMP-activated protein kinase (AMPK) which influences a number of cell metabolism [11].

We previously established four kinds of PEM-resistant mesothelioma with a stepwise increase of PEM concentrations and assayed the resistance with a colony-forming assay [12]. We found that all the resistant cells were not cross-resistant to CDDP and presumed a differential mechanism as to drug resistance of the agents. We selected two kinds of PEM-resistant cells which did not increase expression of TS, DHFR or GARFT in comparison with the respective parent cells [12], and further searched for a possible candidate which might be related with PEM resistance. In this study, we found with a microarray analysis that expression levels of six genes were elevated in two paired cells and investigated a possible role of the candidates in the PEM resistance. We identified one of the genes increased the expression and suggested it as a possible marker for PEM resistance.

## Materials and methods

### Cells and agents

Human mesothelioma cells, NCI-H28, NCI-H226, MSTO-211H and NCI-H2452, and immortalized Met-5A cells of mesothelium origin were purchased from American Type Culture Collection (Manassas, VA, USA), and mesothelioma, EHMES-1 and JMN-1B cells, were provided by Dr. Hironobu Hamada (Hiroshima University, Japan) [13]. PEM-resistant H28-PEM, H226-PEM, 211H-PEM, and H2452-PEM cells were previously established by a stepwise increase of PEM (Eli Lilly, Indianapolis, IN, USA) [12]. Cells were cultured with in RPMI-1640 medium supplemented with 10% fetal calf serum, and confirmed to be negative for mycoplasma. The genotype of *p53* was wild-type in NCI-H28, NCI-H226, MSTO-211H and NCI-H2452 cells but p53 protein of NCI-H2452 cells was truncated [14]. In contrast, the genotype of EHMES-1 and JMN-1B cells was mutated. A769662 (Abcam, Cambridge, UK) and nutlin-3a (Selleck, Houston, TX, USA) were used to stimulate endogenous the AMPK and the p53 pathways, respectively.

### Identification of genes up-regulated in PEM-resistant cells

An aliquot of total RNA was labeled with a fluorescence dye and hybridized with a whole human genome array (44Kx4 ver 2.0, Agilent Technologies, Santa Clare, CA, USA). Expression of respective genes and clustering of the gene expression was analyzed with GeneSpring GX11.5 (Agilent).

### RNA interference

Cells were transfected with small interfering RNA (si-RNA) duplex targeting cardiac ankyrin repeat protein (CARP) (si-RNA-s502326, s502327, s502328) (Thermo Fisher Scientific, Fremont, CA, USA), insulin-like growth factor binding protein-3 (IGFBP3) (si-RNA-s7227, s7228, s7229) (Thermo Fisher Scientific), or nonspecific si-RNA as a control (Thermo Fisher Scientific) using Lipofectamine RNAiMAX according to the manufacturer’s protocol (Thermo Fisher Scientific).

### Reverse transcription-polymerase chain reaction (RT-PCR)

Total RNAs were isolated with TRIzol reagent (Thermo Fisher Scientific) from cells transfected with siRNAs for IGFBP3. First-strand cDNA was synthesized from the RNA preparations using Superscript III reverse transcriptase (Invitrogen, Carlsbad, CA, USA) and amplification of equal amounts of the cDNA was performed with the following primers and conditions: for the *IGFBP3* gene, 5′-GACAGAATATGGTCCCTGCCG-3′ (forward) and 5′-TTGGAAGGGCGACACTGCT-3′ (reverse), and 15 s at 95 °C for denature/45 s at 60 °C for annealing/26 cycles; for the *glyceraldehyde*-*3*-*phosphate dehydrogenase* (*GAPDH*) gene, 5′-ACCACAGTCCATGCCATCAC-3′ (forward) and 5′-TCCACCACCCTGTTGCTGTA-3′ (reverse), and 15 s at 94 °C/15 s at 60 °C/25 cycles.

### Cell viability test

Cells were seeded into 96-well plates (2.8 × 10^3^ cells per well), treated with si-RNA or human recombinant IGFBP3 (Wako, Osaka, Japan) for 24 h and then incubated with PEM for 72 h. Cell viabilities were assessed with a WST-8 kit (Dojindo, Kumamoto, Japan) and the relative viability was calculated based on the absorbance at 450 nm without any treatments (WST assay). GraphPad Grism ver 6.0 (GraphPad Grism Software, San Diego, CA, USA) was used to calculate 50 or 75% inhibitory concentrations (IC_50_ and IC_75_).

### Western blot analysis

Cell lysate was subjected to sodium dodecyl sulfate–polyacrylamide gel electrophoresis. The protein was transferred to a nitrocellulose membrane and was hybridized with antibody against integrin β-3 (Catalog Number: #13166), AMPK (#2532), phosphorylated AMPK (Thr 172) (#2535), phosphorylated p53 (Ser 15) (#9284) (Cell Signaling, Danvers, MA, USA), plasminogen activator inhibitor (#ab20562), a disintegrin and metalloproteinase with thrombospondin motifs 5 (#ab41037) (Abcam), IGFBP3 (#sc-9028), CARP (sc-30181), β-2 adrenergic receptor (#sc-569) (Santa Cruz Biotechnology, Santa Cruz, CA, USA), phosphorylated H2AX (Ser 139) (#613401) (BioLegend, San Diego, CA, USA), p53 (Ab-6, Clone DO-1) and tubulin-α (Clone DM1A) (Thermo Fisher Scientific) as a control followed by an appropriate second antibody. The membranes were developed with the ECL system (GE Healthcare, Buckinghamshire, UK).

### Enzyme-linked immunosorbent assay (ELISA) for IGFBP3

IGFBP3 concentrations in culture supernatants and cell lysate were measured with a human IGFBP3 ELISA kit (R&D Systems, Minneapolis, USA) according to the manufacturer’s instructions. Optical density was measured based on the absorbance at 450 nm using a micro-plate reader.

## Results

### PEM-resistance assayed with the WST assay

**Fig. 1:**
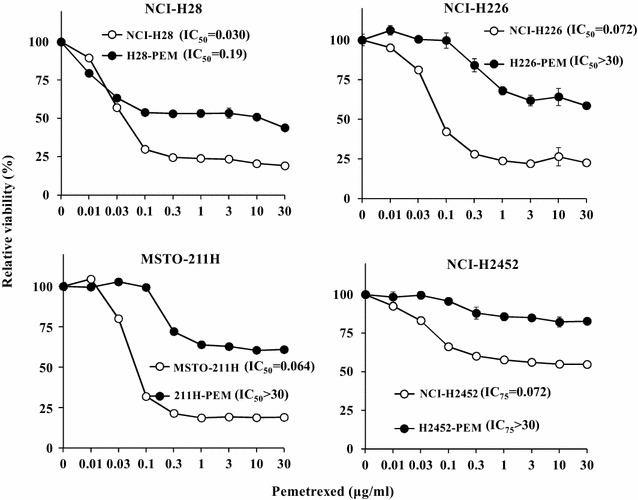
Sensitivity of parent and PEM-resistant cells to PEM. Paired cells of parent and PEM-resistant cells, NCI-H28 and H28-PEM, NCI-H226 and H226-PEM, MSTO-211H and 211H-PEM, and NCI-H2452 and H2452-PEM cells, were treated with various concentrations of PEM for 72 h. Cell viability was measured with the WST assay. Representative data with averages and SE bars (n = 3), and IC_50_ (NCI-H28, NCI-H226, MSTO-211H cells and the respective PEM-resistant cells) and IC_75_ (NCI-H2452 and H2452-PEM cells) values are shown

We established PEM-resistant cells, H28-PEM, H226-PEM, 211H-PEM and H2452-PEM, from respective parent cells, NCI-H28, NCI-H226, MSTO-211H and NCI-H2452, and showed the resistance with a colony-forming assay [12]. We then demonstrated the resistance with the WST assay by measuring relative cell viability after an exposure to various PEM concentrations (Fig. 1). We confirmed that all kinds of the PEM-resistant cells were less sensitive to PEM than their parent cells. NCI-H28, NCI-H229 and MSTO-211H cells showed similar PEM sensitivity but NCI-H2452 cells were relatively resistant to PEM.

### Identification of up-regulated genes in PEM-resistant cells

We previously showed that both 211H-PEM and H2452-PEM cells elevated *TS* and *GARFT* mRNA expression in comparison with respective parent cells, whereas the expression levels of H28-PEM and H226-PEM cells were not elevated or rather lower than those of their parent cells [12]. The *DHFR* transcripts in H28-PEM and H226-PEM cells also decreased compared with the parent cells [12]. We thereby selected two kinds of paired cells, NCI-H28 and H28-PEM, and NCI-H226 and H226-PEM cells, for further analyses to select the genes which elevated the expression greater in PEM-resistant than in the parent cells. We first conducted a microarray analysis which compared gene expression profiles of parent cells with that of PEM-resistant cells in the four kinds of mesothelioma cells (Additional file 1: Fig. S1). We included CDDP-resistant mesothelioma cells in the analyses, which were also established with the same method as the PEM-resistant cells [12]. The analysis detected 21,964-24,509 spots in these parent cells and similar spot numbers were also found in respective CDDP- and PEM-resistant cells. A hierarchical clustering analysis with 19,307 spots revealed that differential expression profiles of respective parent cells were distinctive among these parent cells more than those between individual parent cells and the respective CDDP- or PEM-resistant cells. We therefore screened genes of which the expression were up-regulated in PEM-resistant cells 5 times greater than that in the parent cells, and identified six genes from the two kinds paired cells (Table 1).

**Table 1:** Genes up-regulated in the expression 5 times greater in PEM-resistant cells then in the parent cells

Gene	Ratio of the expression (PEM-resistant cells/parent cells)	
	H28-PEM/NCI-H28	H226-PEM/NCI-H226
Cardiac ankyrin repeat protein (CARP)	6.320	28.949
Adrenergic receptor β-2 (ADRB2)	8.997	21.626
Plasminogen activator inhibitor 1 (PAI1)	9.017	10.212
Integrin β-3 (ITGB3)	10.944	9.041
A disintegrin and metalloproteinase with thrombospondin motifs 5 (ADAMTS5)	7.787	7.233
Insulin-like growth factor binding protein-3 (IGFBP3)	10.861	5.174

Candidate genes listed were confirmed for the differential mRNA expression levels both in paired cells

### CARP and IGFBP3 were differentially expressed in PEM-resistant cells

**Fig. 2:**
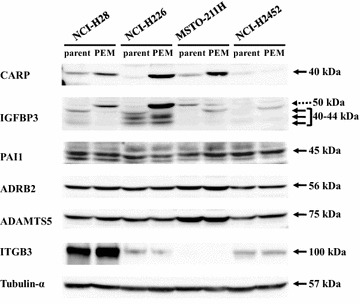
CARP and IGFBP3 were differentially expressed in PEM-resistant cells. Expression of six candidate molecules in parent and PEM-resistant cells (PEM) were examined with Western blot analysis. Cell lysates were probed with antibody as indicated. Tubulin-α was used as a loading control. Dotted arrow indicates a non-specific signal and arrows shows authentic IGFBP3 signals

We examined expression levels of the six up-regulated genes in four paired cells with Western blot analysis (Fig. 2). The analysis showed that CARP expression was augmented in PEM-resistant cells except H2452-PEM cells. IGFBP3 expression showed multiple bands and the intensity in total seemed to increase in all the 4 PEM-resistant cells. The band of the highest molecular weight (50 kDa) however turned out to be a non-specific signal based on experiments with the si-RNA for IGFBP3 and ELISA (see below). The rest of IGFBP3 bands represented several isoforms migrated at molecular weights between 40 and 44 kDa. The IGFBP3 expression in H226-PEM cells could be greater than that in NCI-H226 cells, whereas the expression in H28-PEM cells, showing only a non-specific band at 50 kDa, rather decreased in comparison with that in the parent cells expressing the multiple isoforms. The IGFBP3 level in 211H-PEM and H2452-PEM cells was marginally greater than that of the respective parent cells. In contrast, expression of other molecules, PAI1, ADRB2, ADAMTS5 and ITGB3, was not different between the PEM-resistant and the parent cells. We selected CARP and IGFBP3 based on the differential expression levels and further examined the functions in PEM-resistance. We currently do not know a reason of the discrepant levels between mRNA and protein in respective paired cells.

### Decreased CARP expression did not influence the PEM-resistance

**Fig. 3:**
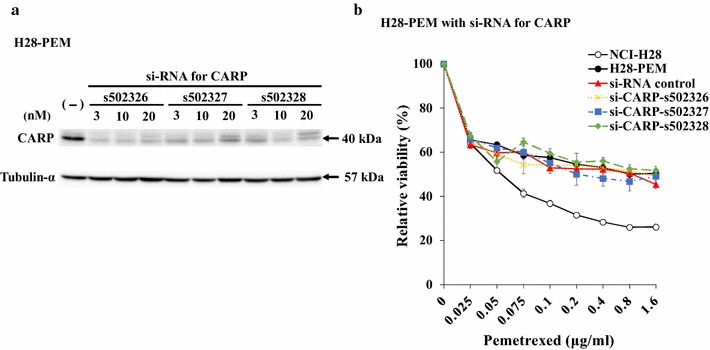
Effects of decreased CARP expression on PEM-resistance. **a** H28-PEM cells were transfected with si-RNA for CARP (three kinds, s502326, s502327 and s502328) at different concentrations and CARP expression levels were examined with Western blot analysis 48 h after the transfection. Tubulin-α was used as a loading control. **b** Cell viability of H28-PEM cells transfected with si-RNA for CARP (10 nM) or control si-RNA (10 nM) and then treated with PEM for 72 h. The cell viability was measured with the WST assay. SE bars are shown (n = 3)

We examined a possible role of CARP in the PEM resistance by down-regulating the expression with si-RNA. We firstly investigated transfection efficiency with different doses of Alexa Fluor red fluorescent control in H28-PEM cells (Additional file 2: Table S1). The fluorescence values showed that 10 nM si-RNA was enough to obtain sufficient efficacy. H28-PEM cells were then treated with three kinds of si-RNA for CARP (s502326, s502327 and s502328) and each kind of si-RNA down-regulated the CARP expression (Fig. 3a). The cells, irrespective of the si-RNA, did not show any changes in their sensitivity to PEM (Fig. 3b). We also tested 221H-PEM cells, expressing CARP greater than parent MSTO-211H cells, and found that the si-RNA treatments did not enhance the PEM sensitivity (Additional file 3: Fig. S2). These results suggested that elevated CARP expression did not contribute to the PEM resistance.

### Production of IGFBP3 was not correlated with the PEM resistance

**Fig. 4:**
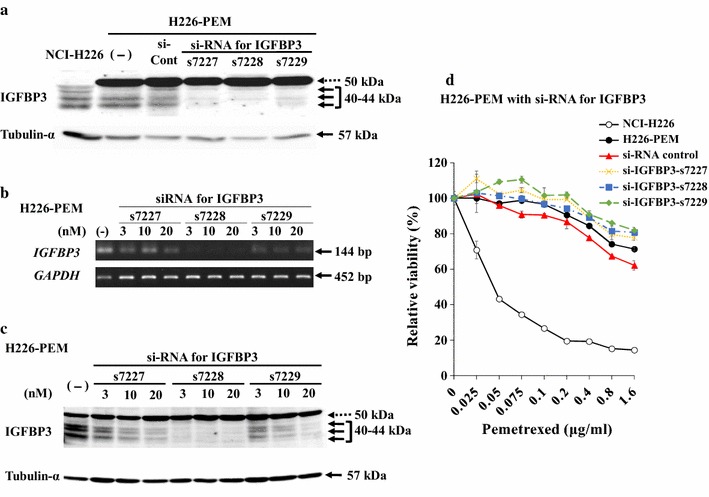
Effects of decreased IGFBP3 expression on PEM-resistance. **a**–**c** si-RNA for IGFBP3 (20 nM) (s7227, s7228 and s7229) or different amounts of si-RNA for IGFBP3 (3, 10 and 20 nM), and control si-RNA (si-Cont) were transfected into H226-PEM cells, and suppression of IGFBP3 expression was confirmed by RT-PCR (**b**) and Western blot analysis (**a**, **c**) 48 h after the transfection. Tubulin-α was used as a loading control. Dotted arrow indicates a non-specific signal and arrows shows authentic IGFBP3 signals. **d** Cell viability of H226-PEN cells transfected with si-RNA for IGFBP3 (3 nM) or control si-RNA (3 nM) and then treated with PEM for 72 h. The cell viability was measured with the WST assay. SE bars are shown (n = 3)

We measured the production of IGFPB3 in culture supernatants and cell lysate with an ELISA kit (Table 2). H226-PEM cells produced IGFBP3 greater than the parent cells in culture supernatants and cell lysate, but H28-PEM cells rather produced less amounts of IGFBP3 compared with the parent cells. These ELISA data were concordant with the expression levels detected with Western blot analysis. We further examined a possible role of IGFBP3 in the drug resistance since IGFBP3 influenced IGF-mediated signaling which was linked with the drug insensitivity [15]. We transfected three kinds of si-RNA for IGFBP3 (s7227, s7228, and s7229) into H226-PEM cells and examined the expression levels with their parent cells with ELISA (Additional file 4: Fig. S3) and Western blot analysis (Fig. 4a). ELISA data showed decreased production of IGFBP3 and Western blot data indicated that the band corresponding the highest molecular weight remained unchanged in the intensity, but lower bands below the highest became significantly weak. We also examined various doses of si-RNA and confirmed down-regulated IGFBP3 transcripts (Fig. 4b). The si-RNA treatments showed that intensity of the lower bands decreased in a dose-dependent manner (Fig. 4c). These si-RNA experiments confirmed that the lower molecular bands but not the highest band corresponded to IGFBP3. We then tested the PEM sensitivity of H226-PEM cells treated with the si-RNA and showed that knocking down of IGFBP3 did not improve susceptibility to PEM (Fig. 4d). We also examined whether recombinant IGFBP3 decreased PEM sensitivity to NCI-H226 cells but the exogenous IGFBP3 did not affect the sensitivity in a dose-dependent manner (Additional file 5: Fig. S4). These data collectively showed that IGFBP3 was not involved in susceptibility to PEM.

**Table 2:** Production of IGFBP3 from mesothelioma cells

Source	Cells	Amounts (ng per 1 × 10^6^ cells)
		(Average ± SE)
Supernatants	NCI-H28	24.01 ± 0.09
	H28-PEM	6.31 ± 0.03
	NCI-H226	100.57 ± 0.18
	H226-PEM	131.10 ± 0.61
Cell lysate	NCI-H28	3.01 ± 0.02
	H28-PEM	1.35 ± 0.01
	NCI-H226	9.56 ± 0.13
	H226-PEM	23.26 ± 0.37

IGFBP3 amounts from supernatants of 2 days-culture or cell lysate were measured with ELISA

The amounts were calculated with a standard IGFBP3 protein and SEs are also shown (n = 3)

### PEM induced up-regulated CARP but not IGFBP3 expression

**Fig. 5:**
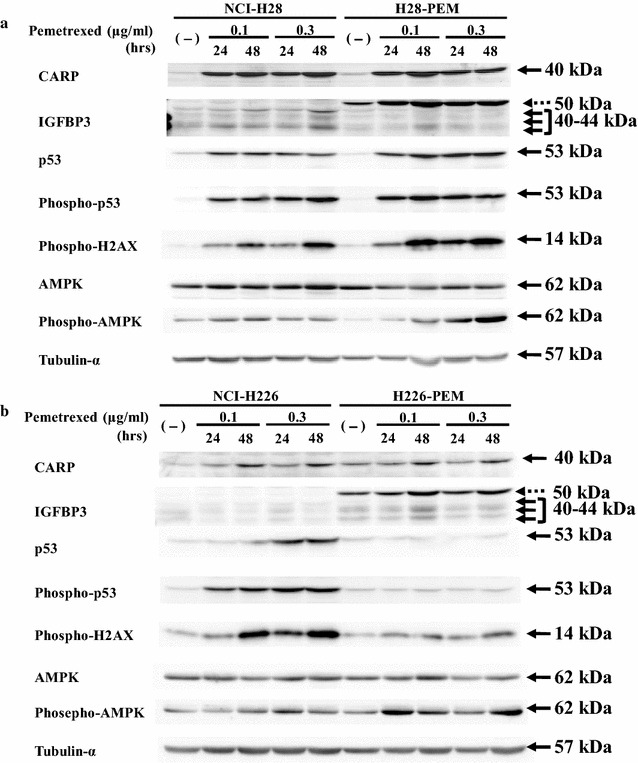
PEM induced differential molecular expression in parent and PEM-resistant cells. Parental and PEM-resistant cells, **a** NCI-H28 and H28-PEM and **b** NCI-H226 and H226-PEM, were treated with PEM (0.1 or 0.3 μg/ml) for 24 or 48 h and the cell lysates were probed with respective kinds of antibody. Exposure times between (**a**) and (**b**) blots were not equal. Tubulin-α was used as a loading control. Dotted arrow indicates a non-specific signal and arrows shows authentic IGFBP3 signals

We examined possible augmentation of CARP and IGFBP3 expression with PEM treatments. We treated paired cells with PEM and investigated the expression with Western blot analysis (Fig. 5). Both NCI-H28 and H28-PEM cells augmented CARP expression, but up-regulation of the IGFBP3 was minimal. NCI-H226 cells and less significantly H226-PEM cells showed increased CARP expression with PEM treatments, but the IGFBP3 expression was minimally or scarcely changed. These data therefore showed that PEM treatments up-regulated CARP but not IGFBP3 expression.

### Augmented p53 contributed to the CARP up-regulation

**Fig. 6:**
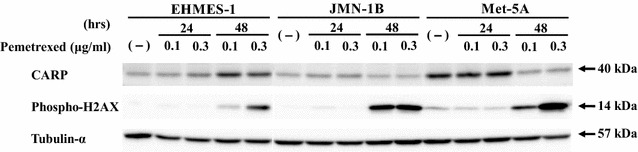
CARP expression in PEM-treated cells mesothelioma with mutated *p53* genotype or immortalized mesothelial cells expressing dominant-negative p53. Cells were in treated with PEM (0.1 or 0.3 μg/ml) for 24 or 48 h. Cell lysates were probed with antibody as indicated. Tubulin-α was used as a loading control

**Fig. 7:**
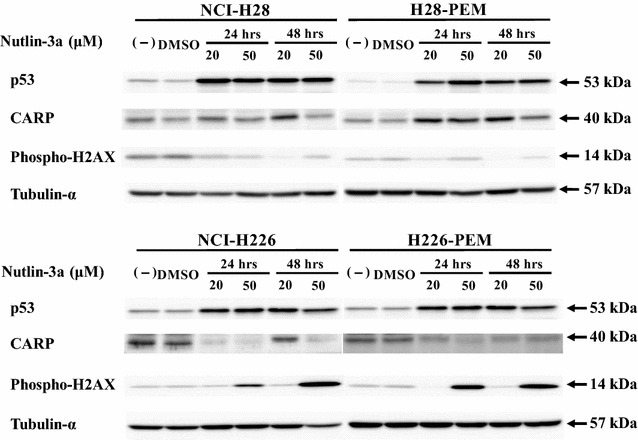
CARP expression in nutlin-3a-treated cells. Parent and the PEM-resistant cells were treated with nutlin-3a or DMSO as a solvent for 24 or 48 h and the cell lysate was probed with antibody as indicated. Tubulin-α was used as a loading control

We investigated a mechanism of the constitutive increase of CARP expression by treating cells with PEM (Fig. 5). We firstly examined p53 expression after PEM treatments in NCI-H28 and NCI-H226 cells, both of which had the wild-type *p53* genotype, and in the PEM-resistant cells. The PEM treatments induced phosphorylation of p53 at serine 15 in NCI-H28, H28-PEM and NCI-226H cells, and consequently the p53 levels were up-regulated in these cells. In contrast, H228-PEM cells did not show the augmented p53 expression or phosphorylated p53, which could be attributable to less sensitivity of the cells to PME-mediated cytotoxicity. We then examined induction of phosphorylated H2AX, a DNA damage marker, and found that the enhanced level in H228-PEM cells was less than that in the other cells which showed the increased expression in a time- and a dose-dependent manner. These data suggested that the up-regulated level of CARP was associated with a degree of DNA damages followed by activation of the p53 pathways. We therefore examined whether the CARP up-regulation was linked with the *p53* genotype. We used mesothelioma cells with mutated *p53* genotype, EHMES-1 and JMN-1B, and mesothelium-derived immortalized Met-5A cells that expressed the SV40 T antigen (Fig. 6). PEM-treated EHMES-1 cells augmented CARP expression levels, but JMN-1B cell and more remarkably Met-5A cells decreased the expression, whereas phosphorylated H2AX levels increased in all the cells upon PEM treatments. These data thereby indicated that PEM-mediated CARP augmentation was not directly dependent on DNA damages. We further examined whether augmentation of endogenous p53 without DNA damages contributed to increased CARP expression. Nutlin-3a inhibited interaction between p53 and MDM2 molecules which mediated ubiquitination of p53 and subsequent p53 degradation [16]. Nutlin-3a-treated cells with the wild-type *p53* therefore increased p53 levels due to inhibiting the p53 degradation processes (Fig. 7). NCI-H28 and H28-PEM cells treated with nutlin-3a increased CARP expression levels, whereas treated NCI-H226 and H226-PEM cells rather decreased the CARP levels except a temporally increased NCI-H226 sample treated at 20 μM for 48 h. Phosphorylated levels of H2AX were dependent on a cell type and a treated dose. Cells originated from NCI-H28 cells increased endogenous p53 without increased H2AX phosphorylation but those from NCI-H226 cells augmented the phosphorylation at 50 μM nutlin-3a treatments. Nutlin-3a was not essentially genotoxic but treatments at a high concentration induced DNA damages in cells of NCI-H226 origin. These data collectively indicated that a way of CARP induction was subjected to cell type difference. Activation of the p53 pathways in NCI-H28 cells was associated with the increased CARP expression but DNA damages was irrelevant. In contrast, neither p53 up-regulation nor DNA damages were linked in cells derived from NCI-H226 cells.

**Fig. 8:**
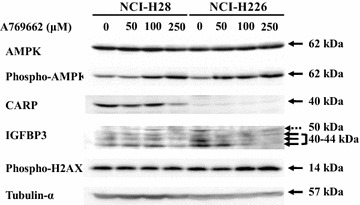
CARP expression in cells treated with an AMPK activating agent. NCI-H28 and NCI-H226 cells were treated with A769662 at various concentrations as indicated for 48 h. The cell lysates were probed with respective antibody. Tubulin-α was used as a loading control

We also examined the effects of PEM on AMPK expression and the phosphorylation (Fig. 5). All the cells including H226-PEM cells increased phosphorylation of AMPK but the AMPK levels remained unchanged, which indicated that PEM stimulated the AMPK signal pathway. These data raised possibility that PEM-mediated augmentation of CARP expression was attributable to activation of the AMPK pathway. We then examined a role of the AMPK activation with an AMPK stimulating agent, A769662 (Fig. 8). NCI-H28 and NCI-H226 cells treated with A769662 increased phosphorylated AMPK, but the CARP expression was not induced or rather decreased. IGFBP3 was not induced by A769662 either. These data showed that activation of AMPK pathway was not involved in the PEM-mediated CARP augmentation.

## Discussion

We showed up-regulated CARP expression in PEM-resistant mesothelioma cells and demonstrated that PEM treatments augmented the CARP expression. The PEM treatments induced DNA damages, up-regulated p53 expression and phosphorylation of AMPK, but the current study indicated that the CARP augmentation was irrelevant to DNA damages or AMPK activation, but was associated with activation of the p53 pathways in some of cells with the wild-type *p53* genotype. Furthermore, we demonstrated that down-regulation of CARP did not influence on PEM sensitivity.

Expression of CARP in human tumors has not well investigated. A previous report showed that clinical specimens of rhabdomyosarcoma expressed the protein at a high frequency since the tumors were derived from CARP-positive striated muscles [17]. In contrast, non-rhabdomyosarcoma expressed CARP at a low frequency [17] and the expression in mesothelioma has not been investigated. CARP physiologically functions not only as a structural component of muscle sarcomere but as a transcriptional co-factor mediating gene expression for muscle stretches [18]. Moreover, the expression in endothelial cells can be linked with wound healing and neovascularization, which may be regulated at the transcriptional level [19]. Regulation of the expression and the functions in non-muscular tissues and tumors can also be distinctive from muscular tissues. A possible relation between CARP expression and drug resistance remained unknown except ovarian cancer [20, 21]. CDDP-resistant ovarian carcinoma cells expressed CARP at a high level and CDDP treatments decreased the CARP expression. Furthermore down-regulation of CARP with the si-RNA increased susceptibility to CDDP [20, 21]. These previous studies collectively suggested that CARP worked for cell survival. In contrast, the present data showed that CARP expression increased upon PEM treatments, but down-regulated CARP was irrelevant to the PEM sensitivity although the CARP expression was up-regulated in PEM-resistant cells. A possible role of CARP in drug resistance can be different among tumors and dependent on chemotherapeutic agents.

The current study showed that PEM treatments increased not only CARP expression but p53, the phosphorylated p53, phosphorylated H2AX and phosphorylated AMPK levels. PEM is a DNA damaging agent and both NCI-H28 and NCI-H226 cells had the wild-type *p53* gene; consequently, PEM stimulated the p53 pathways through a DNA damaging signal. We then examined a possible role of the p53 pathways in augmentation of CARP expression. EHMES-1 cells mutated *p53* genotype increased CARP expression, whereas the expression of JMN-1B cells with the mutated genotype remained unchanged. Furthermore, immortalized Met-5A cells with loss of p53 functions decreased the expression. These data collectively indicate that enhanced expression of CARP was not always associated with activation of the p53 pathways. On the other hand, PEM induced DNA damages, evidenced by phosphorylated H2AX expression, in all the cells. We therefore examined the CARP expression with nutlin-3a, which induced p53 under a non-genotoxic condition, and found that CARP expression was enhanced in NCI-H28 and the PEM-resistant cells. Nutlin-3a at a high concentration however induced DNA damages in NCI-H226 and the PEN-resistant cells probably due to off-target effects. Nevertheless, the cells treated with a low concentration of nutlin-3a augmented p53 levels without DNA damages. CARP expression levels in NCI-H226 and the PEM-resistant cells remained unchanged in most of the cases irrespective of p53 up-regulation and DNA damages. These data indicated that the CARP up-regulation was linked with activation of the p53 pathways but not with DNA damages in some of mesothelioma cells. Interestingly, a previous study showed that CARP induced p53 expression and p53 in turn activated CARP transcription [22]. The present study did not fully support the reciprocal induction between CARP and p53 since nutlin-3a differentially influenced on CARP expression in NCI-H28 and NCI-H226 cells. The present study rather indicated that p53-induced CARP up-regulation was dependent on cell types. Expression of CARP is also regulated by a number of factors including GADD153 and SMAD4 [19, 23]. These regulatory factors are further influenced by various conditions and their expression are controlled by different mechanisms. GADD153 is in fact induced by DNA damages and down-regulated CARP [23]. The cell type difference of CARP induction can therefore be attributable to complexity of the gene regulations.

Pemetrexed also stimulated the AMPK pathway that was evidenced by phosphorylated AMPK. PEM-treated cells accumulated ZMP, an intermediated AMP analogue, activated the APMK [10, 11]. We used an AMPK activating agent, A769662, to examine a possible involvement of AMPK activation but the AMPK agonist did not augment CARP expression. The current study consequently showed that AMPK activation was not involved in up-regulation of CARP expression.

We established PEM-resistant cells but a possible mechanism of the drug insensitivity was not well characterized. Comparison of parent and the PEM-resistant cells after PEM treatments revealed less DNA damages induced in H226-PEM cells than NCI-H226 cells. Phosphorylation of p53 was also insignificant in PEM-treated H226-PEM cells, whereas nutlin-3a-treated H226-PEM cells augmented p53 and phosphorylated H2AX to a similar degree as the parent cells. These data indicated that one of the mechanisms for PEM resistance in H226-PEM cells was linked with an impaired system of sensing PEM-mediated DNA damages. Our previous study showed that acquired PEM insensitivity was irrelevant to several enzyme activities which mediated PEM metabolism and the current investigation implied possible correlation between the resistance and CARP up-regulation. A precise mechanism of the augmented CARP expression remains yet unclear and the increased expression was not related with PEM resistance. A continuous stimulation of the AMPK system by PEM may result in suppression of the mammalian target of rapamycin pathway, one of the targets of AMPK, and subsequently decrease DNA synthesis, which can be linked with PEM resistance. We therefore speculate that constitutive AMPK activation contributes to PEM insensitivity and presume that investigation on this association will be one of the next research targets.

Mesothelioma often develops into being resistant to the first-line agent after a few courses of chemotherapy and no second-line regimen is yet known. A biomarker for the PEM-resistance is therefore not clinically useful at this moment and moreover clinical specimens from the patients who fail to respond are usually unavailable. Nevertheless, early detection of PEM-resistance, although such a marker not currently known, will be beneficial for the patients to evaluate their current therapeutics and be valuable when shifting into a possible second-line agent that is hopefully available in future.

## Conclusions

We demonstrated that CARP expression was elevated in PEM-resistant mesothelioma cells and suggested that the up-regulated expression was a candidate marker for PEM resistance in mesothelioma although down-regulated CARP expression did not influence the PEM sensitivity. The augmented CARP expression was not always linked with immediate cellular responses but activation of the p53 pathways, depending on cell types, was involved in the CARP up-regulation.

## Additional files

**Additional file 1: Figure S1.** A heat map of microarray analyses. Expression profiles among parent, CDDP- and PEM-resistant cells of 4 kinds of mesothelioma cells were analyzed with the whole human gene expression microarray (Agilent Technology, Santa Clara, CA, USA). A cell name with CDDP indicates CDDP-resistant cells.

**Additional file 2: Table S1.** Transfection efficacy of si-RNA.

**Additional file 3: Figure S2.** Cell viability of 211H-PEM cells transfected with si-RNA for CARP (10 nM) or control si-RNA (10 nM) and then treated with PEM for 72 hrs. The cell viability was measured with the WST assay. SE bars are shown (n=3).

**Additional file 4: Figure S3.** Inhibited secretion of IGFBP3 with si-RNA. H226-PEM cells were transfected with si-RNA for IGFBP3 (20 nM) (s7227, s7228 and s7229) or control si-RNA (si-Cont), and the culture supernatants were assayed with ELISA. We measured optical density at 450 nm and used 10 ng rhIGFBP3 as a positive control. Average and SE bars are shown (n=3).

**Additional file 5: Figure S4.** Influence of recombinant human IGFBP3 (rhIGFBP3) on PEM-resistance. NCI-H226 cells were treated with different doses (12.5, 25 and 50 ng/ml) of rhIGFBP3 for 24 hrs, then treated with PEM for further 72 hrs. Cell viability was measured using the WST assay. SE bars are shown (n=3).

## Abbreviations

AMPK: AMP-activated protein kinase
CARP: cardiac ankyrin repeat protein
CDDP: cisplatin
DHFR: dihydrofolate reductase
ELISA: enzyme-linked immunosorbent assay
GARFT: glycinamide ribonucleotide formyltransferase
IGFBP3: insulin-like growth factor binding protein-3
PEM: pemetrexed
RT-PCR: reverse transcription-polymerase chain reaction
TS: thymidylate synthase
ZMP: 5-amino-4-imidazolecarboxamide ribotide
