# Supplementary figures and images for: Augmented expression of cardiac ankyrin repeat protein is induced by pemetrexed and a possible marker for the pemetrexed resistance in mesothelioma cells
Source: Cancer Cell Int. 2017 Dec 11;17:120. doi: 10.1186/s12935-017-0493-8 (PMC5725641; doi:10.1186/s12935-017-0493-8)

**Figure S1**

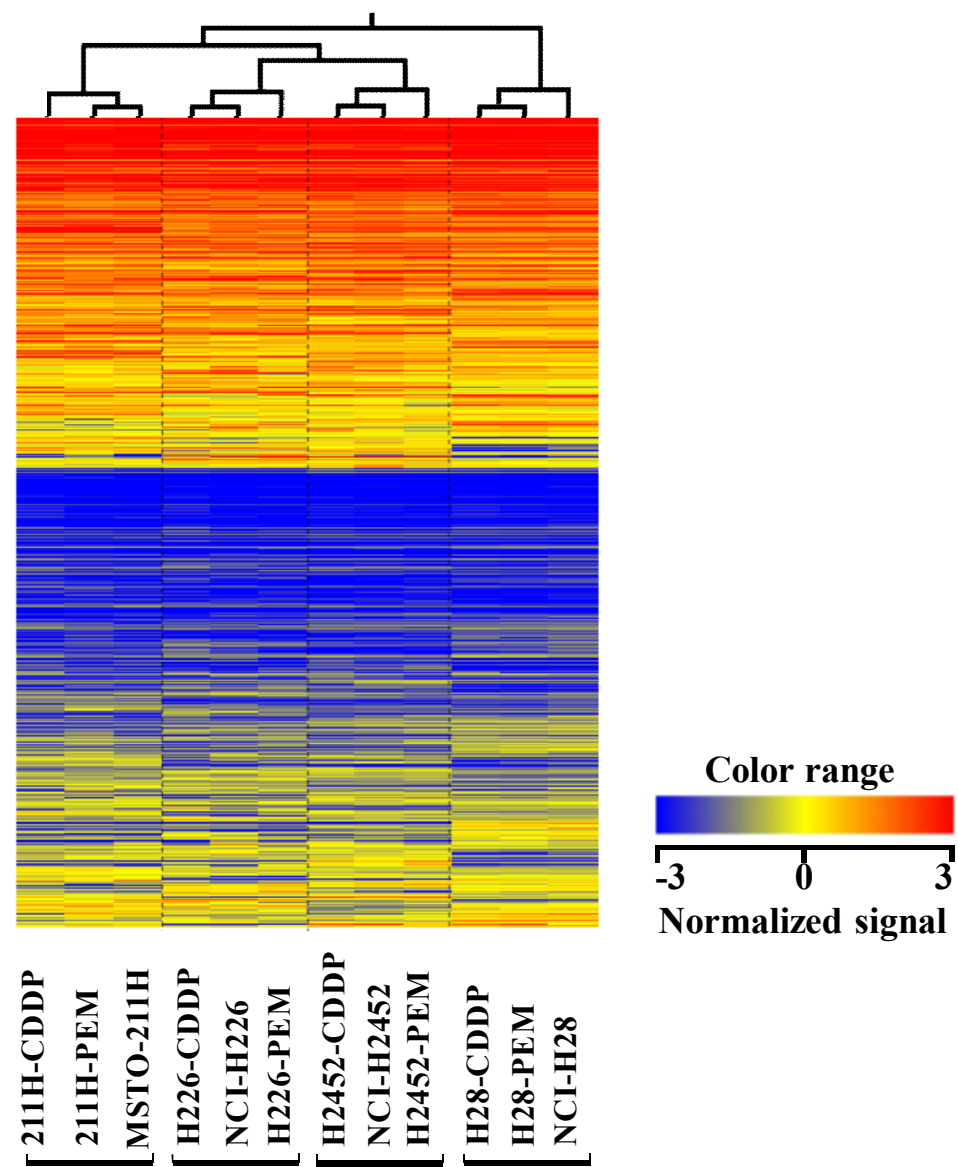

Supplement: Supplementary file 1 — Additional file 1: Figure S1. A heat map of microarray analyses. Expression profiles among parent, CDDP- and PEM-resistant cells of 4 kinds of mesothelioma cells were analyzed with the whole human gene expression microarray (Agilent Technology, Santa Clara, CA, USA). A cell name with CDDP indicates CDDP-resistant cells. [file 12935_2017_493_MOESM1_ESM.pdf]

**Figure S2**

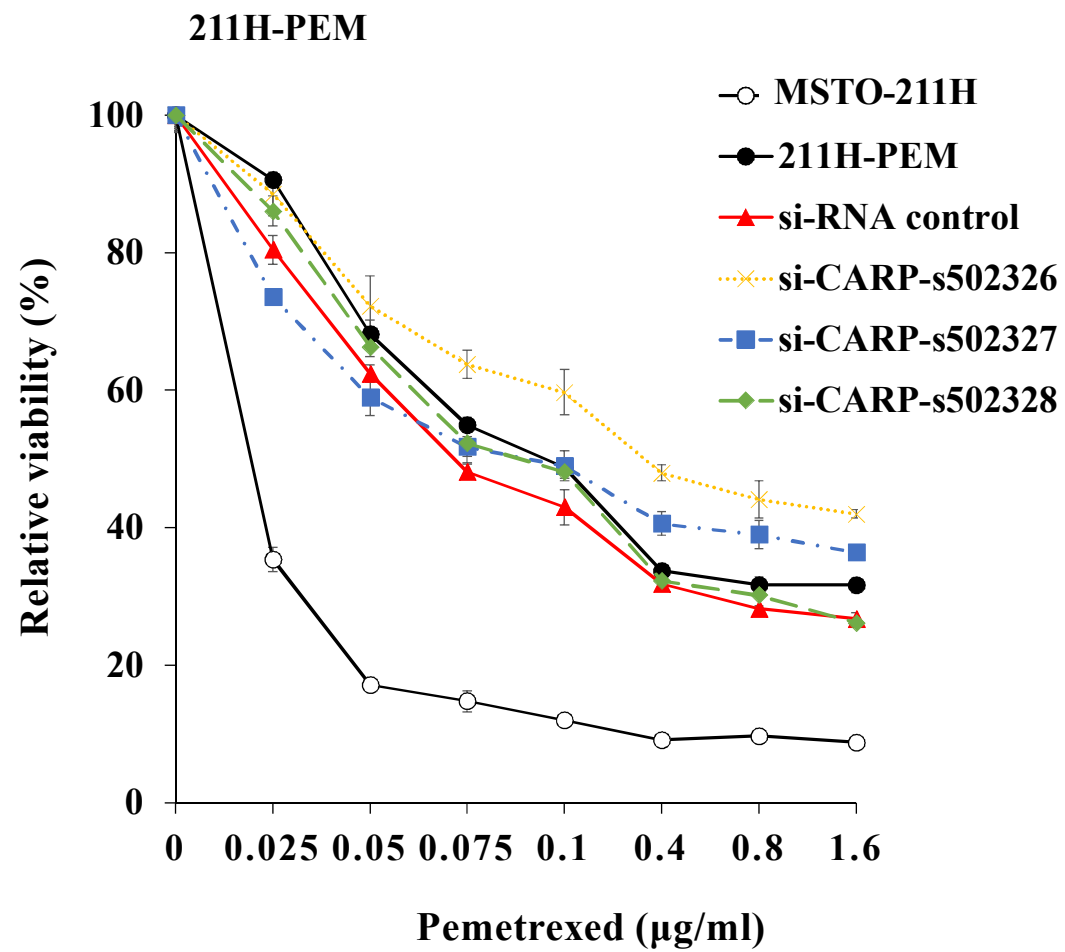

Supplement: Supplementary file 3 — Additional file 3: Figure S2. Cell viability of 211H-PEM cells transfected with si-RNA for CARP (10 nM) or control si-RNA (10 nM) and then treated with PEM for 72 hrs. The cell viability was measured with the WST assay. SE bars are shown (n=3). [file 12935_2017_493_MOESM3_ESM.pdf]

**Figure S3**

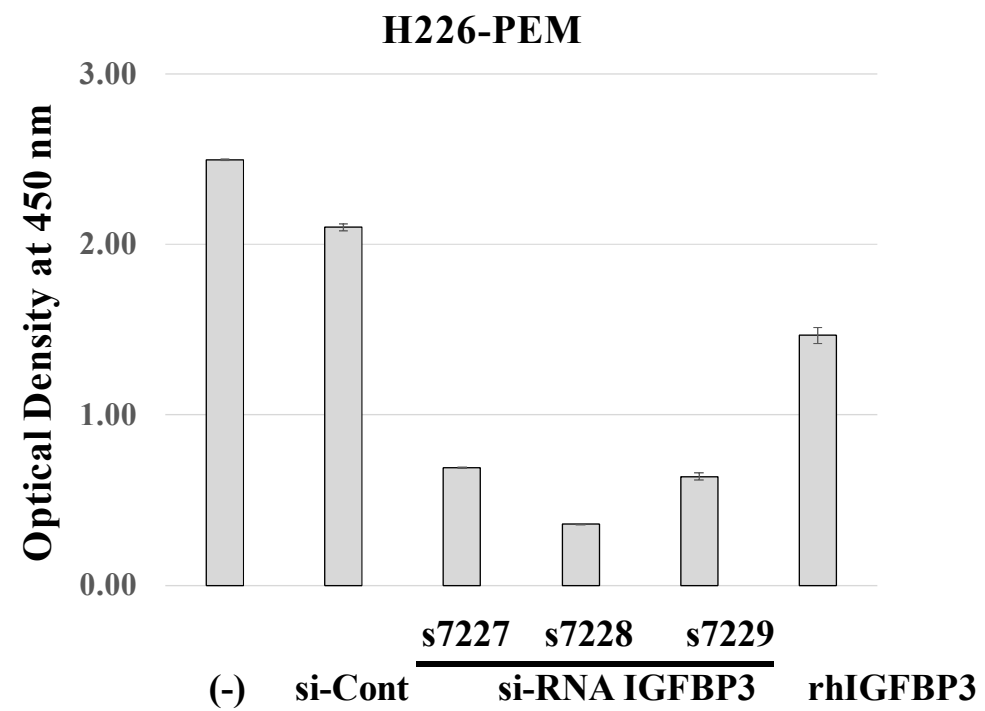

Supplement: Supplementary file 4 — Additional file 4: Figure S3. Inhibited secretion of IGFBP3 with si-RNA. H226-PEM cells were transfected with si-RNA for IGFBP3 (20 nM) (s7227, s7228 and s7229) or control si-RNA (si-Cont), and the culture supernatants were assayed with ELISA. We measured optical density at 450 nm and used 10 ng rhIGFBP3 as a positive control. Average and SE bars are shown (n=3). [file 12935_2017_493_MOESM4_ESM.pdf]

Figure S4

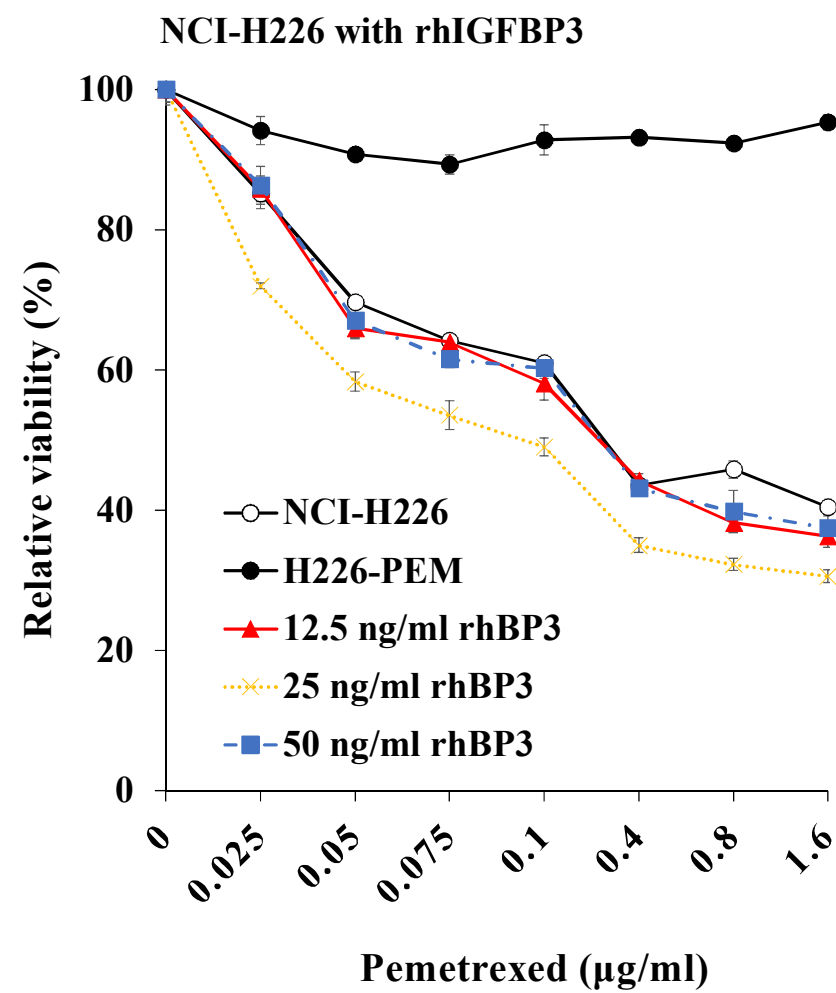

Supplement: Supplementary file 5 — Additional file 5: Figure S4. Influence of recombinant human IGFBP3 (rhIGFBP3) on PEM-resistance. NCI-H226 cells were treated with different doses (12.5, 25 and 50 ng/ml) of rhIGFBP3 for 24 hrs, then treated with PEM for further 72 hrs. Cell viability was measured using the WST assay. SE bars are shown (n=3). [file 12935_2017_493_MOESM5_ESM.pdf]
