# Supplementary material for: Augmented expression of cardiac ankyrin repeat protein is induced by pemetrexed and a possible marker for the pemetrexed resistance in mesothelioma cells
Source: Cancer Cell Int. 2017 Dec 11;17:120. doi: 10.1186/s12935-017-0493-8 (PMC5725641; doi:10.1186/s12935-017-0493-8)
Supplement: Supplementary file 2 — Additional file 2: Table S1. Transfection efficacy of si-RNA. [file 12935_2017_493_MOESM2_ESM.docx]

**Table S1** Transfection efficacy of si-RNA

Concentrations of dye (nM) Percent red positive cells (Average + SE)

0 0

1 13.80 + 0.01

3 32.08 + 0.02

10 80.78 + 0.01

50 90.24 + 0.01

Various doses of Alexa Fluor red fluorescent control (Thermo Fisher Scientific) was transfected into H28-PEM cells and efficiency of the transfection was assessed by the fluorescence detected with the Tali image-based cytometer (Thermo Fisher Scientific). Cells whose fluorescence was greater than the brightest 5% of uninfected cells were judged as positively stained. Averages and SE values are shown (n=3).
